# The reverse mode of the Na^+^/Ca^2+^ exchanger contributes to the pacemaker mechanism in rabbit sinus node cells

**DOI:** 10.1038/s41598-022-25574-8

**Published:** 2022-12-17

**Authors:** Noémi Tóth, Axel Loewe, Jozefina Szlovák, Zsófia Kohajda, Gergő Bitay, Jouko Levijoki, Julius Gy. Papp, András Varró, Norbert Nagy

**Affiliations:** 1 Department of Pharmacology and Pharmacotherapy, Albert Szent-Györgyi Medical School, University of Szeged, Dóm tér 12, P.O. Box 427, Szeged, 6720 Hungary; 2 Institute of Biomedical Engineering, Karlsruhe Institute of Technology (KIT), Karlsruhe, Germany; 3 ELKH-SZTE Research Group of Cardiovascular Pharmacology, Szeged, Hungary; 4 Orion Pharma, Espoo, Finland

**Keywords:** Cardiology, Cardiovascular biology

## Abstract

Sinus node (SN) pacemaking is based on a coupling between surface membrane ion-channels and intracellular Ca^2+^-handling. The fundamental role of the inward Na^+^/Ca^2+^ exchanger (NCX) is firmly established. However, little is known about the reverse mode exchange. A simulation study attributed important role to reverse NCX activity, however experimental evidence is still missing. Whole-cell and perforated patch-clamp experiments were performed on rabbit SN cells supplemented with fluorescent Ca^2+^-tracking. We established 2 and 8 mM pipette NaCl groups to suppress and enable reverse NCX. NCX was assessed by specific block with 1 μM ORM-10962. Mechanistic simulations were performed by Maltsev–Lakatta minimal computational SN model. Active reverse NCX resulted in larger Ca^2+^-transient amplitude with larger SR Ca^2+^-content. Spontaneous action potential (AP) frequency increased with 8 mM NaCl. When reverse NCX was facilitated by 1 μM strophantin the Ca^2+^_i_ and spontaneous rate increased. ORM-10962 applied prior to strophantin prevented Ca^2+^_i_ and AP cycle change. Computational simulations indicated gradually increasing reverse NCX current, Ca^2+^_i_ and heart rate with increasing Na^+^_i_. Our results provide further evidence for the role of reverse NCX in SN pacemaking. The reverse NCX activity may provide additional Ca^2+^-influx that could increase SR Ca^2+^-content, which consequently leads to enhanced pacemaking activity.

## Introduction

The SN AP is characterized by its slow diastolic depolarization (DD) phase starting from the maximal diastolic potential (MDP) and ending at the AP threshold of about − 40 mV. It was suggested that the decaying delayed rectifier potassium current (I_X1_ or later called I_Kr_)^1^ and the cAMP-dependent hyperpolarization activated funny-current^2^ has a major role forming the DD. This mechanism of pacemaking, driven by transmembrane ion channels with Hodgkin-Huxley kinetics was termed later as “membrane clock” (M clock). Further studies which identified rhythmic, spontaneous subsarcolemmal Ca^2+^ releases (LCR) generated by the sarcoplasmic reticulum (SR) via ryanodine receptors during the DD (“Ca^2+^ clock”) have challenged the dominant role of membrane clock in the spontaneous automaticity of the SN^3–5^. After intense debate regarding the underlying mechanism of spontaneous SN pacemaking, a large body of evidence proved that the M clock and Ca^2+^ clock are functionally tightly coupled, so the two mechanisms form the latest concept of SN automaticity which is called the coupled clock system. This coupling is based on numerous time-, voltage- and Ca^2+^-dependent mechanisms^6,7^ including a major role of the L-type Ca^2+^ current (I_CaL_) that “resets” and ”refuels” the Ca^2+^ clock^6^.

The crucial role of the (forward/inward) NCX in the SN pacemaking as part of the Ca^2+^ clock now is firmly established, and represents a fundamental mechanism of spontaneous pacemaking, however little is known about the reverse NCX in SN. Several computational NCX models have not considered a significant reverse NCX in SN cells^8–13^. To the best of our knowledge, the first reported model regarding SN cells which attributed a significant role to the reverse mode was proposed by Maltsev et al. in 2013, who claimed that Ca^2+^ influx via reverse NCX could be functionally important since its contribution to refilling the sarcoplasmic reticulum is almost as large as the contribution of I_CaL_^14^. This modelling prediction has important implications since it suggested yet unexplored and novel mechanisms contributing to the pacemaking system.

However, the direct experimental validation of these interesting in silico results were hampered so far because of the lack of selective NCX inhibitors, i.e. the absence of a NCX blocker without major effects on I_CaL_. ORM-10962 is a novel and fully selective NCX inhibitor without any effect on the other currents at a concentration of 1 μM where ORM-10962 causes about 80% inhibition of both modes (forward and reverse) of NCX^15–17^.

Therefore, the aim of this study was (i) to experimentally investigate the possible existence of reverse NCX in SN cells and (ii) to explore its probable functional role in SN pacemaking mechanism.

## Methods

All experiments were conducted in compliance with the *Guide for the Care and Use of Laboratory Animals* (USA NIH publication No 85-23, revised 1996) and conformed to Directive 2010/63/EU of the European Parliament. The protocols were approved by the Review Board of the Department of Animal Health and Food Control of the Ministry of Agriculture and Rural Development, Hungary (XIII./1211/2012). Animal studies were carried out in compliance of ARRIVE guidelines.

### Animals

The measurements were performed on spontaneously beating SN cells obtained from sinus node tissue of young New Zealand white rabbits (Innovo Ltd.) from both genders weighing 2.0–2.5 kg.

### Experimental groups

In this study, our aim was to establish two experimental groups throughout the study: one with active reverse mode and the other with blunted reverse exchange. In order to achieve this, the pipette solutions contained 8 mM NaCl and 2 mM NaCl, respectively. 8 mM NaCl were used to approximate the physiological Na^+^ level of the cells, while in the other group, the pipette solution was reduced to 2 mM NaCl aiming to suppress the reverse mode of the NCX without completely abolishing the exchanger function.

### Cell isolation

Isolated single SN cells were obtained by enzymatic dissociation as described previously^18^. Rabbits were sacrificed by concussion after intravenous administration of 400 IU/kg heparin. The chest was opened and after the quick removal of the heart, it was placed into an isolation solution containing in mM: 135 NaCl, 4.7 KCl, 1.2 KH_2_PO_4_, 1.2 MgSO_4_, 10 HEPES, 4.4 NaHCO_3_, 10 glucose, 1.8 CaCl_2_ (titrated to pH 7.2 with NaOH). The heart was mounted on a 60 cm high modified Langendorff column and perfused with oxygenated isolation solution at 37 °C. First, the blood was washed out (3–5 min), then the heart was perfused with nominally Ca^2+^-free solution until the heart stopped contracting (4–5 min). The enzymatic digestion was performed by perfusion with the same isolation solution supplemented with 1.8 mg/ml (260 U/ml) collagenase (type II, Worthington) and 33 µM CaCl_2_. After 13–14 min, the heart was taken off the cannula. The right atrium of the heart was cut and the SN region was excised and cut into small pieces. The strips were placed into the enzyme free isolation solution containing 1 mM CaCl_2_ and equilibrated at 37 °C for 15 min. The cells were separated by filtering through a mesh and were stored at room temperature.

### Measurement of NCX current with voltage-ramp protocol

As a standard pharmacological approach, a voltage-ramp protocol was used to measure the NCX current and to verify the differences in the two experimental groups containing 2 mM or 8 mM Na_pip_ and to investigate the effectiveness of the selective NCX inhibitor ORM-10962. From a holding potential of − 40 mV, the membrane was depolarized to 30 mV with a slope of 0.7 V/s, then hyperpolarized to − 70 mV. The reverse mode was calculated at + 25 mV while the forward operation was calculated at − 60 mV, both during the downhill phase of the current. The pipette solution contained (in mM): 125 CsCl, 20 TEACl, 5 MgATP, 10 HEPES, 8 or 2 NaCl, titrated to pH 7.2 with CsOH. The free intracellular Ca^2+^ was buffered to ~ 100 nM (by using an appropriate mixture of EGTA and Ca^2+^ calculated by using MaxChelator software) to approximate a normal diastolic Ca^2+^ value. The composition of the external solution was: 135 mM NaCl, 10 mM CsCl, 0.33 mM NaH_2_PO_4_, 10 mM TEACl, 1 mM MgCl_2_, 10 mM glucose, 10 mM HEPES, 1 mM CaCl_2_, 20 µM ouabain, 50 µM lidocain, 1 µM nisoldipin, titrated to pH 7.4.

### Measurement of Ni^2+^-sensitive current under predefined AP command

During the voltage clamp experiments from Figs. 2, 3, 4 and 5, SN cells were paced using a previously recorded, canonical AP waveform. This AP waveform was obtained by average of 10 independent APs under perforated patch clamp conditions. The parameters of the AP command were: *cycle length*: 410 ms, *maximal diastolic potential*: − 57 mV, *overshoot*: 24 mV, *action potential duration*: 180 ms, *diastolic depolarization slope*: 0.124 mV/ms.

In the case of experiments demonstrated in Fig. 2, the NiCl_2_ sensitive current under the predefined AP waveform was measured. The extracellular solution contained: 135 mM NaCl, 10 mM CsCl, 0.33 mM NaH_2_PO_4_, 10 mM TEACl, 1 mM MgCl_2_, 10 mM glucose, 10 mM HEPES, 1.8 mM CaCl_2,_ 0.2 mM BaCl_2_, 20 µM ouabain, 50 µM lidocain, 1 µM nisoldipine, 1 µM mibefradil, titrated to pH 7.4. The intracellular solution contained (in mM): 125 CsCl, 20 TEACl, 5 MgATP, 10 HEPES and 10 EGTA titrated to pH 7.2 with CsOH, and 2 or 8 mM NaCl was added respectively.

### Measurements of Ca^2+^-transients and caffeine-response under predefined AP command

The compositions of the applied solution in experiments depicted in Figs. 3, 4 and 5 were the following: external solution containing 135 mM NaCl, 10 mM CsCl, 0.33 mM NaH_2_PO_4_, 10 mM CsCl, 10 mM TEACl, 1 mM MgCl_2_, 10 mM HEPES, 10 mM glucose, 1 mM CaCl_2_, 20 µM ouabain, titrated to pH 7.4. The intracellular solution contained (in mM): 125 CsCl, 5 MgATP, 20 TEACl, 10 HEPES titrated to pH 7.2 with CsOH.

During these measurements, the intracellular Ca^2+^ was unbuffered allowing Ca^2+^ transients (CaT) that were monitored by Fluo-4 AM (5 µM) fluorescent dye. The isolated SN cells were loaded with the dye for 20 min at room temperature in darkness. Fluorescence measurements were performed as it was described in our previous study^18^. Experiments were carried out on the stage of an Olympus IX 71 inverted fluorescence microscope. The dye was excited at 480 nm and the emitted fluorescence was detected at 535 nm. Optical signals were sampled at 1 kHz and recorded by a photon counting photomultiplier (Hamamatsu, model H7828). Amplitudes of the Ca^2+^ transients were calculated as differences between systolic and diastolic values. To measure Ca^2+^ changes, the cells were damaged by a patch pipette at the end of the experiment to obtain maximal fluorescence (F_max_). Ca^2+^ was calibrated using the following formula: K_d_(F − F_min_)/(F_max_ − F). The K_d_ of the Fluo-4 AM was set to 335 nM.

### Action potential measurements from single cells by current clamp configuration

Spontaneous APs were measured by perforated or whole cell patch clamp configuration from spontaneously beating rabbit SN cells. The perforated patch measurements followed the method of Lyashkov et al.^19^. The membrane potential was recorded in current clamp configuration using a gap-free acquisition protocol. Normal Tyrode’s solution was used, containing (in mM): 144 NaCl, 0.4 NaH_2_PO_4_, 4 KCl, 0.53 MgSO_4_, 1.8 CaCl_2_, 5.5 glucose and 5 HEPES, titrated to pH 7.4. The patch pipette solution contained (in mM): 120 K-gluconate, 2.5 NaCl, 2.5 MgATP, 2.5 Na_2_ATP, 5 HEPES, 20 KCl, titrated to pH 7.2 with KOH. 35 µM β-escin was added to the pipette solution to achieve the membrane patch perforation. The cells were loaded with Fluo-4 AM to measure intracellular Ca^2+^ changes following the method that was described here previously (“Measurements of Ca^2+^-transients and caffeine-response under predefined AP command” Section).

When whole cell configuration was used, the pipette solution was almost identical with the composition used above, it contained (in mM): 120 K-gluconate, 2.5 MgATP, 2.5 K_2_ATP, 5 HEPES, 20 KCl supplemented with 2 mM NaCl or 8 mM NaCl accordingly to 2 mM [Na^+^]_pip_ and 8 mM [Na^+^]_pip_ groups, titrated to pH 7.2 with KOH. The cells were filled with Fluo-4AM.

Membrane voltage or ionic currents were obtained by using an Axoclamp 1-D amplifier (Molecular Devices, Sunnyvale, CA, USA) connected to a Digidata 1440A (Molecular Devices, Sunnyvale, CA, USA) analogue–digital converter. The membrane voltage was recorded by Clampex 10.0 (Molecular Devices, Sunnyvale, CA, USA).

The parameters of the APs were calculated as follows:

- Maximum diastolic potential (MDP) was calculated as the most negative potential reached before the AP depolarization.
- Take off potential (TOP) defined as the voltage measured at the time when the voltage derivative exceeded 0.5 mV/ms.
- The slope of depolarization was calculated as the mean voltage derivative of the AP between MDP and take off potential.
- Action potential duration (APD) defined as the time interval between TOP and the next MDP.
- Cycle length was measured between the peaks of two consecutive APs.
- All experiments in this study were performed at 37 °C.

### Statistics

Normal distribution of the data was verified by using Shapiro–Wilk test. In this study, we used hierarchical analysis: the technical replicates obtained from the same heart were averaged providing n = 1. Therefore, the experimental number ‘n’ refers the number of hearts used. Thus, all experiments can be considered independent. For the experiments, 3–5 sinus node cells were used from each rabbit. Statistical significance (*p* < 0.05) was assessed using Student’s t-test, or repeated measures ANOVA. Data are presented as mean ± S.E.M.

### Modeling

To mechanistically underpin our experimental findings, we conducted numerical simulations using the Maltsev et al. minimal model^14^. The minimal model is a variant of the Maltsev et al. 2009 model neglecting I_CaT_, I_Ks_, I_to_, I_sus_, I_f_, I_st_, I_bNa_, I_NaK_ and I_bCa_^20^. Intracellular sodium and potassium concentrations are considered as constant in this model. The choice of model was motivated by a focus on fundamental mechanisms and the comparability to the experimental setting where potassium currents and I_f_ were inhibited. We based our simulations on model #1, parameter set #4 as defined in^14^. The code was obtained from the CellML model repository and integrated using Matlab’s ordinary differential equation solver *ode15s* (The Mathworks, Natick, MA, USA). All model codes are available in the Supplement.

## Results

### Experimental validation of 2 and 8 mM [Na]_pip_ groups

In the first set of experiments conventional NCX-ramp protocol was used to study the absence and presence of a reverse current in 2 and 8 mM [Na]_pip_ conditions***, ***respectively. We also compared the effect of 1 µM ORM-10962 on the NCX current in the presence of 2 and 8 mM [Na]_pip_ to exclude any Na-dependent action of ORM.

**Figure 1:**
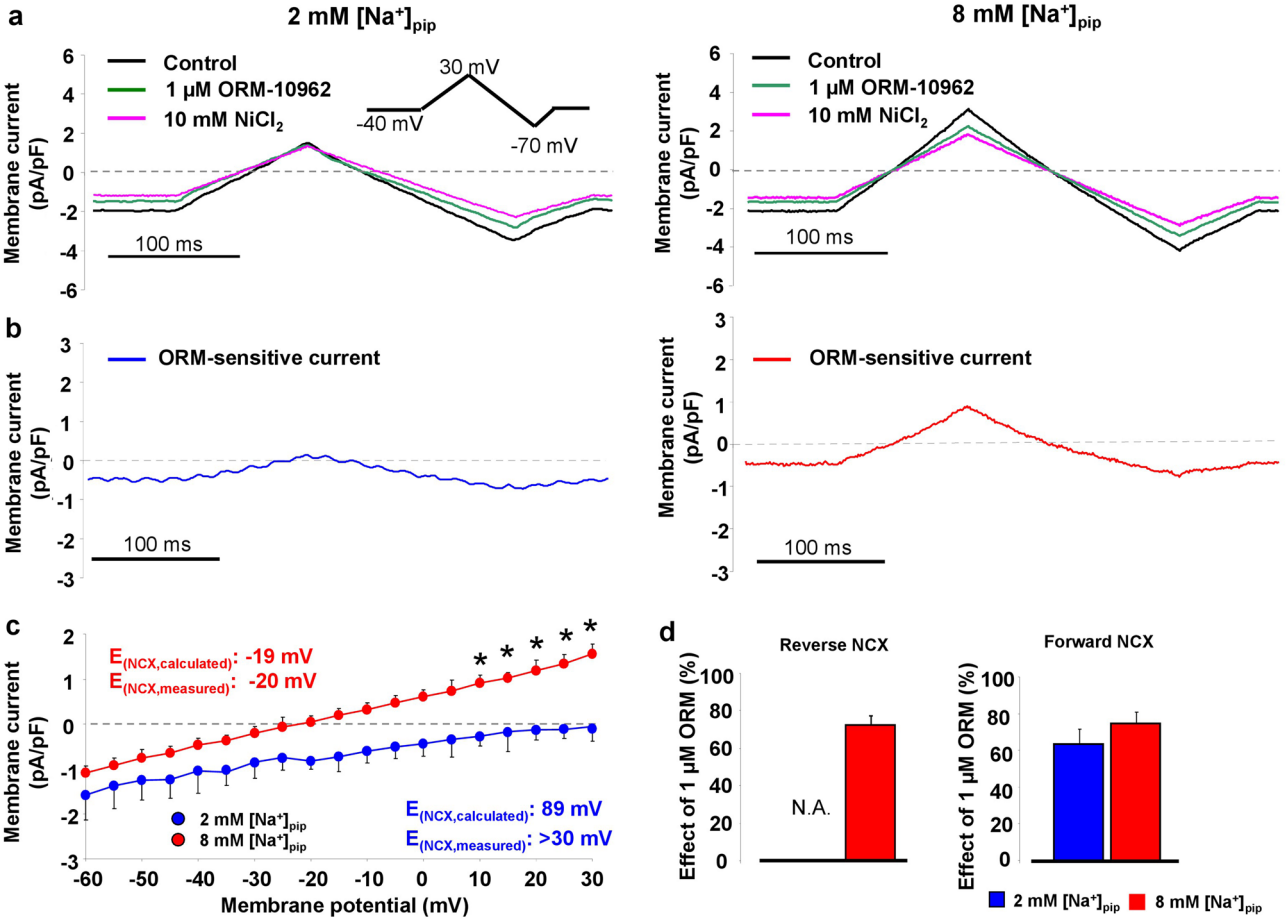
Characterization of experimental groups and the effect of 1 μM ORM-10962. Representative original current traces of control (black), 1 μM ORM-10962 (green) and 10 mM NiCl_2_ (pink) obtained under a conventional voltage ramp protocol (see inset). The intracellular Ca^2+^ was set to ~ 100 nM, K^+^ and Ca^2+^ currents were inhibited. In the presence of 2 mM [Na]_pip_ (panel **a**, left side, repeated measures ANOVA) we did not find neither ORM- nor NiCl_2_-sensitive currents in the outward direction. In contrast, the outward component is clearly observable when 8 mM NaCl was used in the pipette (panel ** a**, right side, repeated measures ANOVA). Panel (**b**) represents the ORM-10962 sensitive currents obtained from the experiment demonstrated in panel A. Current–voltage diagram (panel **c**) of the NiCl_2_-sensitive current demonstrates the lack of reverse current when 2 mM NaCl was employed in the internal solution (blue graph, independent t-test). Panel (**d**) illustrates comparison of the ORM-effect between experimental groups, and we found that 1 μM ORM-10962 similarly reduces the forward component of the NCX independent from the Na_i_ level that may exclude pharmacological interactions between the applied Na_i_ and ORM-10962 (independent t-test). Data shown as mean ± SEM, n = 7, * means *p* < 0.05.

After registration of the control current (Fig. 1a, black traces), 1 µM ORM-10962 was applied (Fig. 1a, green traces) and finally 10 mM NiCl_2_ (Fig. 1a, pink traces) was used to dissect the total NCX current. Panel b illustrates the ORM-10962 sensitive currents obtained from this experiment. In the case of 2 mM [Na]_pip_ group (left side, blue curve) the current lacks the outward component. The total NCX current was calculated as a difference of the control and the Ni^2+^-insensitive current (Fig. 1c). As Fig. 1a left panel, and Fig. 1b (blue graph) indicate, we did not find outward current component in the presence of 2 mM [Na]_pip_. In contrast, Fig. 1a right panel demonstrates that 1 µM ORM-10962 and 10 mM NiCl_2_ dissected significant amount of outward current when 8 mM NaCl was applied in the pipette (repeated measures ANOVA). A marked difference between the reversal potentials of the experimental groups was also observed: in the 8 mM [Na]_pip_ group the reversal potential well approximates the calculated reversal potential, indicating the existence of a thermodynamical possibility for the reverse mode operation (panel c, independent t-test).

Indeed, the reversal potential in the 2 mM [Na]_pip_ group is obviously far from the calculated value indicating that the intracellular Na^+^ level sensed by the NCX was larger than the pipette Na^+^ concentration. However, the NCX current was found to be negative from − 60 to + 30 mV in these experiments. Since our experiments were carried out within this range, we considered that reverse NCX is approximately absent in our experiments when 2 mM [Na]_pip_ was employed. It is important to note that the inward component of the current (i.e., forward mode) in the presence of 8 mM [Na]_pip_ did not differ from the current measured in the presence of 2 mM [Na]_pip_ indicating that the operation of the forward modes was identical between groups.

Since we planned to use ORM to inhibit NCX (in the later part of the study), we considered important to exclude any possible Na^+^-dependent effect of the ORM prior to these experiments. The effect of ORM-10962 was calculated as a ratio between the total current (control vs 10 mM NiCl_2_) and the ORM-inhibited fraction (control vs 1 µM ORM-10962). As Fig. 1d illustrates, we found no significant difference of the ORM effects between the 2 and 8 mM [Na]_pip_ groups (2 mM fwd mode: 63.5 ± 8%, n = 6; 8 mM fwd mode: 74.5 ± 6%, n = 7; independent t-test). Since we did not observe reverse NCX in the presence of 2 mM NaCl, we could not quantify ORM effect in this case. The effect of ORM on the reverse mode in the presence of 8 mM [Na]_pip_ was: 72.5 ± 5%, n = 7.

In the presence of 8 mM [Na]_pip_, both ORM-10962 and NiCl_2_ dissected a notable outward current from the control, which may reflect the potential existence of reverse NCX that may be Na-dependent and increases as the membrane potential becomes more positive.

Therefore, based on these results, we consider the 8 mM [Na]_pip_ group as an experimental condition where the reverse mode is active, and the 2 mM [Na]_pip_ group as having suppressed or no reverse exchange activity.

**Figure 2:**
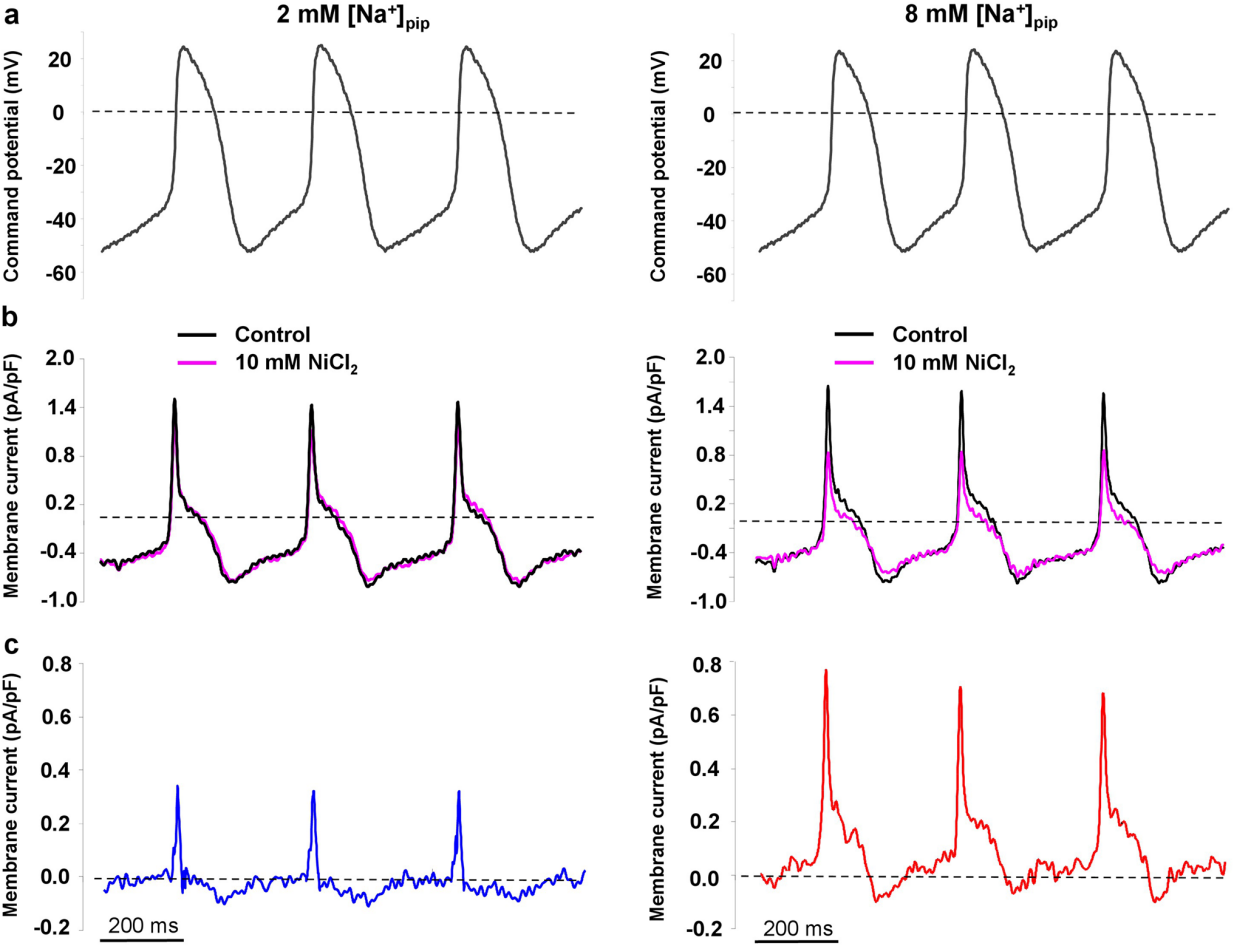
Characterization of the NCX current as a Ni^2+^-sensitive current under a canonical SN action potential (panel **a**). The intracellular Ca^2+^ was buffered with 10 mM EGTA, K^+^ and Ca^2+^ currents were inhibited. Left side of the Figure demonstrates 2 mM [Na]_pip_, right side shows 8 mM [Na]_pip._ Panel (**b**) illustrates the control (black) and the NiCl_2_-treated currents (pink) and panel (**c**) depicts the subtracted currents.

In the next set of experiments, we aimed to investigate whether the reverse NCX current develops under a SN action potential. In these experiments, a previously recorded canonical SN AP waveform was used (Fig. 2a) as command potential (see Methods for parameters), and the intracellular Ca^2+^ was buffered by using 10 mM EGTA. The K^+^-currents, I_CaL_, I_CaT_, I_Na/K_ were inhibited during the experiments. After recording the control current, the NCX current was estimated by application of 10 mM NiCl_2_ (Fig. 2b) in order to fully inhibit the NCX. After current subtraction (Fig. 2c) in the presence of 2 mM [Na]_pip_, an outward current carrying 0.33 ± 0.3 pC was found (n = 5, capacitance: 51 ± 1 pF). In the presence of 8 mM [Na]_pip_, this net outward charge transport was significantly larger (2.1 ± 0.3 pC, n = 7, *p* < 0.05, capacitance: 52 ± 1 pF, independent t-test). This value is slightly smaller than the one predicted by the Maltsev–Lakatta model (2.45 pC).

It is important to note that under this setting the Ca^2+^ release was blocked due to Ca^2+^-channel inhibition and intracellular Ca^2+^ buffering. It was necessary, since 10 mM NiCl_2_ (concentration needed to complete inhibition of NCX) also suppresses the I_CaL_, which would seriously contaminate the results. Therefore, this method allows estimating the total carried charges through the reverse mode, however, lacks the Ca^2+^ release, which is a crucial component of the NCX driving force. Thus, 1 µM ORM-10962 was used to explore the reverse NCX in the presence of Ca^2+^ release (Supplementary Fig. 1). Results indicate the existence of the reverse NCX as an outward ORM-sensitive current in the very beginning of the action potential, however, ORM-10962 only partially inhibit the NCX. Similarly, to the results with NiCl_2_, the outward component of the ORM-sensitive current was absent when 2 mM NaCl was applied in the patch pipette.

### Without reverse NCX activity the Ca^2+^ transient is smaller

**Figure 3:**
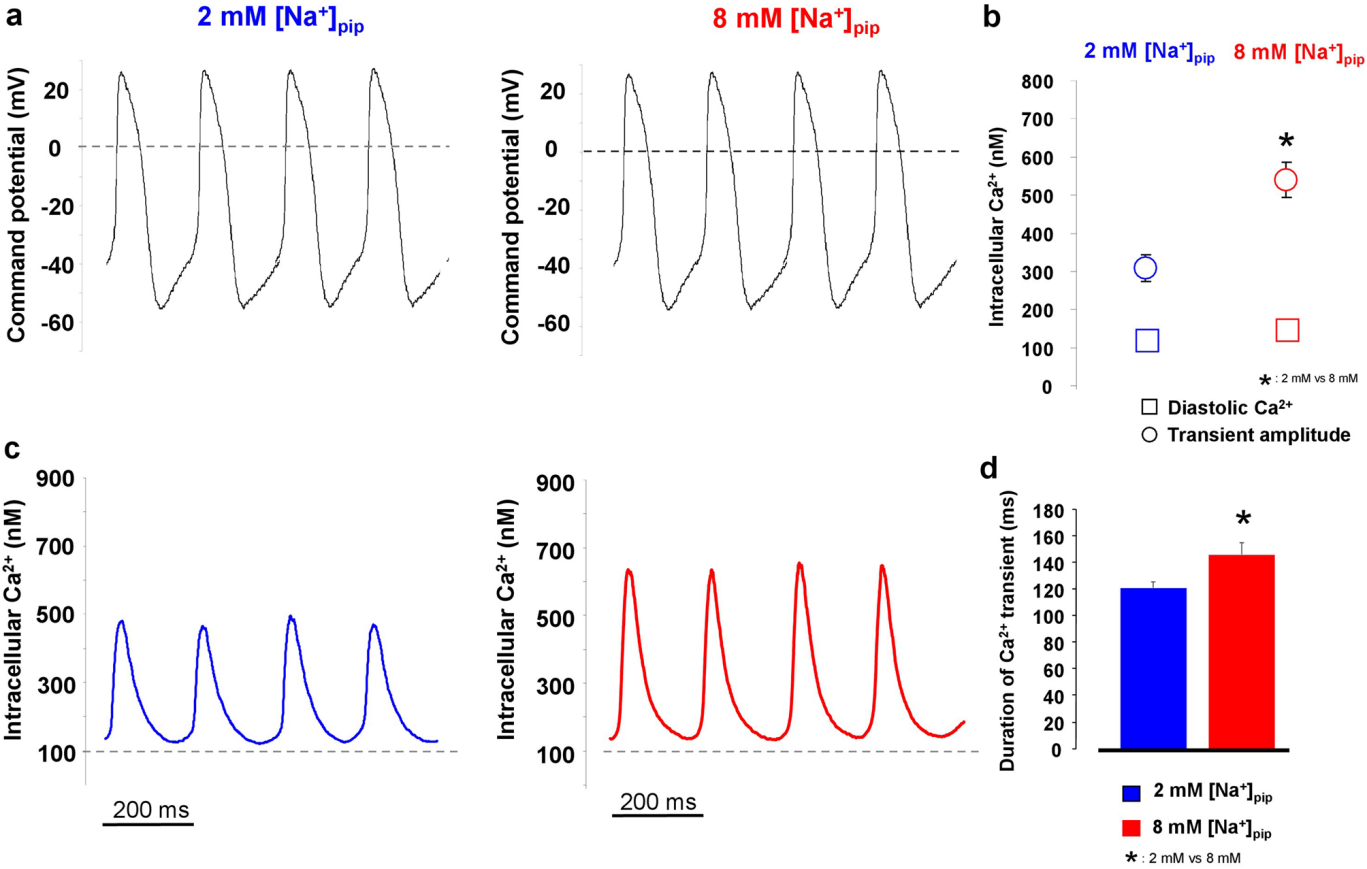
An estimation of Ca^2+^ transients under a canonical SN action potential command potential in the presence of 2 mM (left) and 8 mM (right) NaCl in the pipette solution. The compositions of the extra and intracellular solutions ensured that K-currents, I_f_, and Na/K pump were inhibited during these measurements. The intracellular Ca^2+^ was unbuffered. Panel (**a**) represents a canonical AP command potential. The amplitude of the parallel measured Ca^2+^ transients (panel **b** and **c**) was larger in the presence of 8 mM [Na]_pip_ compared to 2 mM [Na]_pip_ group while the diastolic Ca^2+^ remained unchanged (panel **c** *refers to the difference of transient amplitudes between 2 and 8 mM [Na]_pip_ and means *p* < 0.05). Similarly, the half relaxation time of the transient decay was slower when 8 mM [Na]_pip_ was applied (panel **d** *refers to the half relaxation time in comparison between 2 and 8 mM [Na]_pip_ groups, and means *p* < 0.05). Data shown as mean ± SEM, n = 14–14, p value was calculated by independent t-test.

In the next set of experiments, we aimed to demonstrate the possible functional consequence of the active reverse NCX on the Ca^2+^ transient magnitude. In these experiments, we used again the canonical AP waveform (Fig. 3a) as command potential and nisoldipine and EGTA were omitted from the pipette solution. During control recordings we compared the diastolic Ca^2+^ level and amplitude of the steady-state Ca^2+^ transients between 8 and 2 mM [Na]_pip_ groups (Fig. 3b). It was found that with active reverse NCX the Ca^2+^ transients exerted higher level of amplitude than with no or supressed reverse NCX (2 mM [Na]_pip_: 308 ± 37 nM, n = 14 vs 8 mM [Na]_pip_: 539 ± 52 nM, n = 14; *p* < 0.05, independent t-test). The diastolic Ca^2+^ level did not change (2 mM [Na]_pip_: 117 ± 14 nM, vs 8 mM [Na]_pip_: 149 ± 24 nM, n = 14–14; *p* < 0.08, independent t-test; Fig. 3b,c). The transient relaxation time measured at 50% of the amplitude was significantly longer in the presence of active reverse exchange (2 mM [Na]_pip_: 121 ± 6 ms vs 8 mM [Na]_pip_: 146 ± 9 ms; n = 14–14, *p* < 0.05; independent t-test; Fig. 3d).

### In the presence of active reverse mode the SR Ca^2+^ content is increased

**Figure 4:**
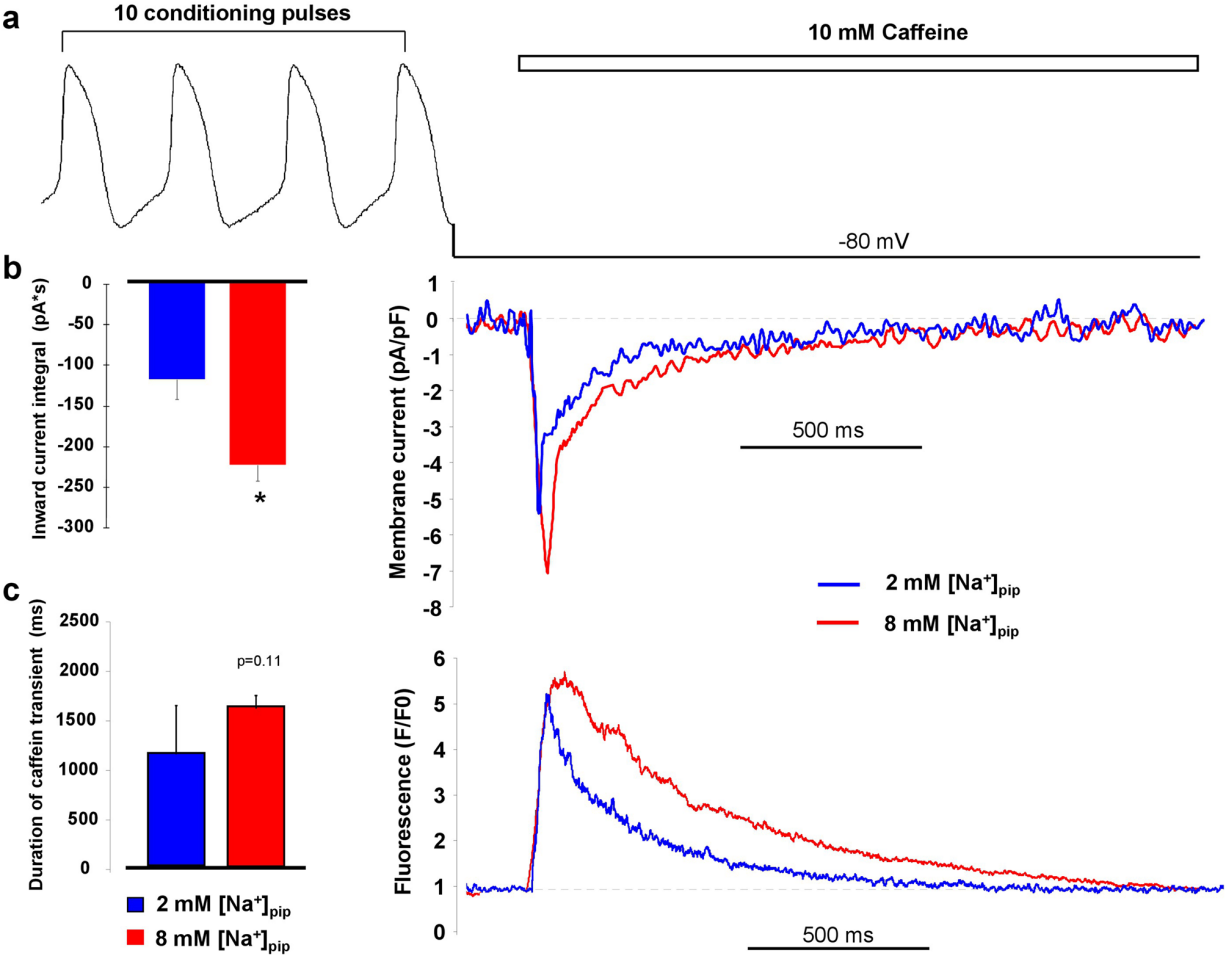
Measurement of the SR Ca^2+^ content by rapid application of 10 mM caffeine. Caffeine was applied after 10 consecutive conditioning pulses to reach a steady-state load of the SR Ca^2+^ content (panel **a**). Panel (**b**) illustrates original caffeine induced inward current, while panel (**c**) illustrates the Ca^2+^ transients in the presence of 2 (blue trace) and 8 mM (red trace) pipette Na^+^. We found larger SR Ca^2+^ content in presence of 8 mM pipette Na^+^ (independent t-test, panel **b**). In contrast, we did not find statistically significant difference between the half-relaxation times of the Ca^2+^ transients (independent t-test, panel **c**). Data shown as mean ± SEM, n = 11 (panel **b**) and n = 17 (panel **c**), *means *p* < 0.05.

The larger Ca^2+^ transient amplitude in the presence of active reverse NCX suggests increased SR Ca^2+^ content. In order to address this question experimentally, we measured the SR Ca^2+^ content by rapid application of 10 mM caffeine. Prior to caffeine administration, 10 consecutive AP commands were applied to reach a steady state Ca^2+^ level of the SR. During caffeine flush, the membrane potential was kept constantly at − 80 mV. The SR Ca^2+^ content was estimated by calculating the integral of the inward current in response to caffeine. As Fig. 4b indicates we found significantly larger SR Ca^2+^ content when reverse mode is active (8 mM [Na]_pip_: − 226.9 ± 20.5 pA*s, n = 11; 2 mM [Na]_pip_: − 121.5 ± 24.2 pA*s, n = 11; *p* < 0.05, independent t-test). The duration of the caffeine-induced Ca^2+^ transient at 50% of the amplitude did not change between the groups (Fig. 4c) of 8 mM [Na]_pip_ (1.59 ± 0.18 s, n = 17) and 2 mM [Na]_pip_ (1.2 ± 0.16 s, n = 16).

### The activity of reverse NCX improves the “recovery” of Ca^2+^ transients after caffeine application

It was hypothesized that the functional role of the reverse NCX could be further demonstrated during SR Ca^2+^ refilling after a caffeine application. Thermodynamical considerations dictate, when SR is empty, the initially small Ca^2+^ releases cause a negative shift of the NCX reversal potential. This may provide a large thermodynamical driving force for the reverse operation during the early phase of refilling. Therefore, an additional Ca^2+^ influx through the reverse NCX may improve the intracellular Ca^2+^ gain compared to cells where reverse exchange is blunted. This question was addressed by reapplication of the consecutive AP commands immediately after 10 mM caffeine application. During voltage command the Ca^2+^ transients were monitored, and the change of diastolic Ca^2+^ level and Ca^2+^ transient amplitude were analysed. Since the absolute values of both the diastolic Ca^2+^ and Ca^2+^ transient amplitudes were generally decreased after the application of caffeine, we normalized both variables for the values obtained during the first pulses.

**Figure 5:**
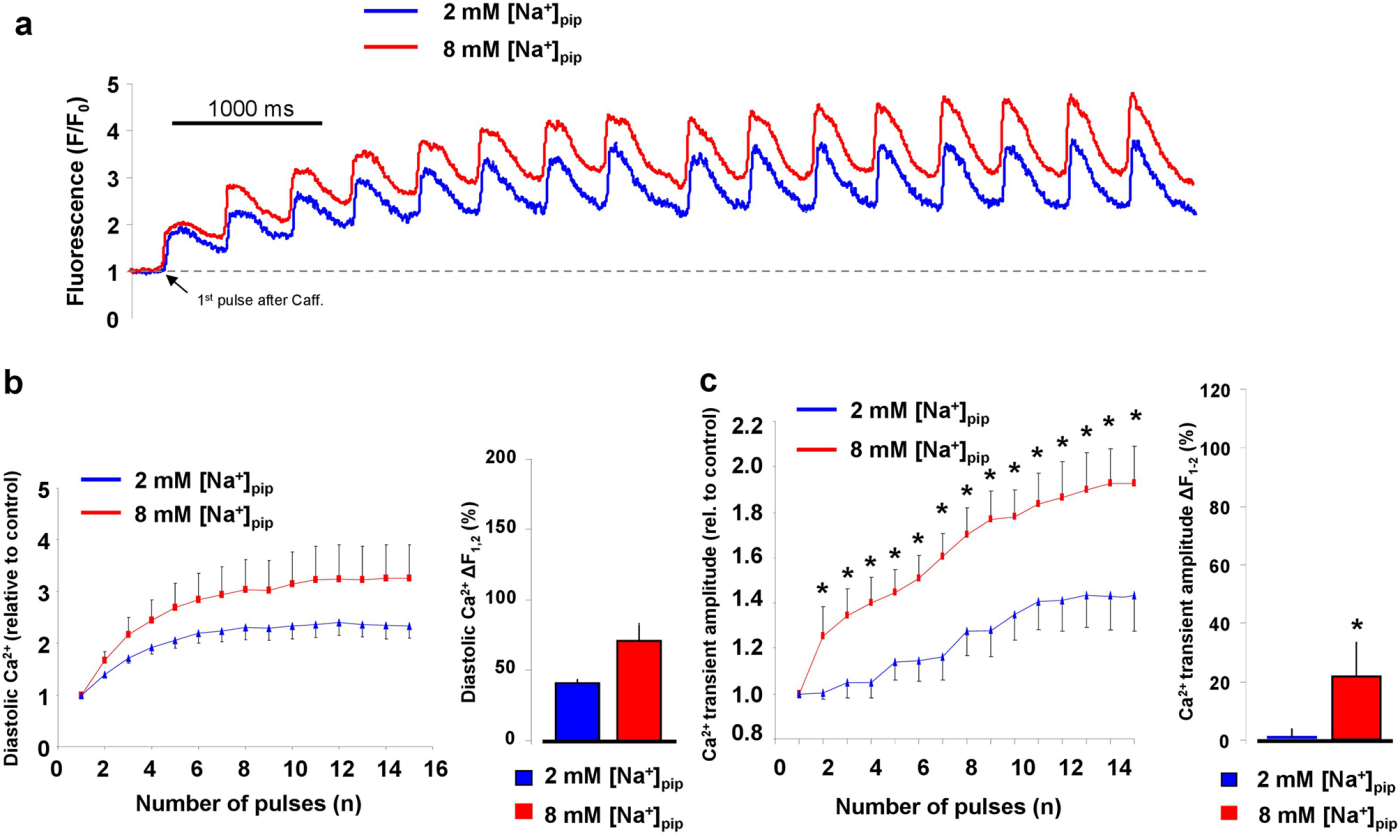
Evaluation of the Ca^2+^-transients after application of 10 mM caffeine. The step-by-step increasing Ca^2+^ transients (panel **a**) reached their steady-state value after the 10th stimulus where the diastolic Ca^2+^ transient (panel **b**) was not changed while the transient amplitude (panel **c**) was higher in the presence of 8 mM pipette NaCl (red graphs). The bar graph in panel (**c**) indicates faster change of transient amplitude between 1st and 2nd beats when pipette Na^+^ was adjusted to 8 mM while in the case of diastolic Ca^2+^ level it was found identical refilling kinetics between 2 (blue column) and 8 (red column) pipette NaCl (bar graph, panel **b**). Independent t-test, *means *p* < 0.05.

The diastolic Ca^2+^ level was analysed after the establishment of the steady-state (at pulse No14) while the amplitude was analysed in all pulses. In agreement with Ca^2+^ transient experiments presented in Fig. 3b, the diastolic Ca^2+^ did not show difference under steady state (14th: 3.2 ± 0.6 vs 2.2 ± 0.8; n = 13–13, independent t-test, Fig. 5b). In contrast, larger Ca^2+^ transient amplitudes were found from the 2nd pulse when reverse mode operated (2nd: 1.25 ± 0.1 vs 1.00 ± 0.03, n = 13–15 respectively, *p* < 0.05, independent t-test; Fig. 5c).

As an indicator of the speed of SR Ca^2+^ refilling, we analysed the change of Ca^2+^ levels between consecutive pulses. Between pulse No1 and No2 the diastolic Ca^2+^ level remained unchanged (66.4 ± 14% vs 40.1 ± 5%, n = 13–13, independent t-test), but the Ca^2+^ transient amplitude (27.7 ± 11.9% vs 0.24 ± 2.1%, n = 11–15, *p* < 0.05, independent t-test) exerted faster increase during refilling (Fig. 5b–c, bar graphs) in the presence of active reverse mode.

### Active reverse mode enhances pacemaker activity

**Figure 6:**
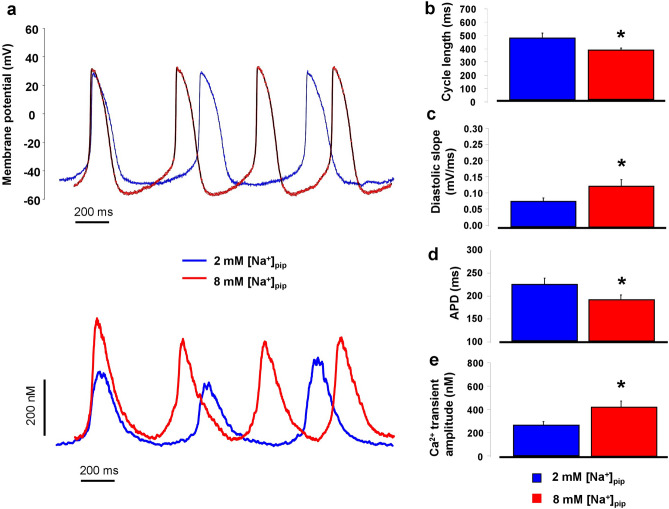
Comparison of spontaneous AP (panel** a**, upper traces) and CaT characteristics (panel **a**, lower traces) between 2 mM (blue traces) and 8 mM (red traces) [Na]_pip_ groups, measured by whole-cell patch clamp technique. Bar graphs indicate that the cycle length of spontaneous APs (panel **b**) was shorter, the diastolic slope (panel **c**) was steeper, the APD (panel **d**) was shorter and CaT amplitude (panel** e**) was larger in the 8 mM [Na]_pip_ group (red columns) compared to 2 mM [Na]_pip_ group (blue columns). Data shown as mean ± SEM, n = 8–8, independent t-test, *means *p* < 0.05.

In order to address the functional importance of reverse mode in spontaneous pacemaking we compared the AP and CaT characteristics between 2 and 8 mM [Na]_pip_ groups obtained using the whole cell patch clamp configuration. The whole cell configuration was selected to provide identical experimental conditions as in previous experiments. As Fig. 6 demonstrates the AP cycle length was shorter when reverse mode was active (i.e.: in the presence of 8 mM [Na]_pip_; 369 ± 15 ms *vs* 463 ± 38 ms; *p* < 0.05, n = 8–8, Fig. 6a–b). In line with this, the slope of diastolic depolarization was steeper when reverse mode was active (0.12 ± 0.02 mV/ms vs 0.07 ± 0.01 mV/ms; *p* < 0.05, n = 8–8, Fig. 6c). The APD was shorter in the 8 mM [Na]_pip_ group (189 ± 3 ms vs 232 ± 11 ms; *p* < 0.05, n = 8–8, Fig. 6d). The Ca^*2*+^ transient amplitude was larger in the presence of 8 mM [Na]_pip_ (420 ± 52 nM vs 250 ± 22 nM; p < 0.05, n = 8–8, Fig. 6e). No change was found in the MDP (8 mM [Na]_pip_: − 55 ± 3 mV, 2 mM [Na]_pip_: − 49 ± 2 mV; *p* = 0.1, independent t-test).

In order to further validate AP data, perforated patch experiments were also performed with 2 and 8 mM NaCl in the pipette. The cycle length, APD and diastolic slope changed similarly between groups as was observed under experiments with the whole cell configuration (Supplementary Fig. 2 and Table 1).

**Table 1:** Comparison of AP data measured by whole cell and perforated patch clamp configurations.

Applied configuration	Whole cell		Perforated patch	
	2 mM [Na^+^]pip	8 mM [Na^+^]pip	2 mM [Na^+^]pip	8 mM [Na^+^]pip
Cycle length (ms)	463 ± 38	369 ± 15*	478 ± 31	392 ± 25*
APD (ms)	232 ± 11	189 ± 3*	226 ± 17	179 ± 11*
Diastolic slope (mV/ms)	0.07 ± 0.01	0.12 ± 0.02*	0.05 ± 0.01	0.098 ± 0.01*
MDP (mV)	− 49 ± 2	− 55 ± 3	− 52 ± 3	− 53 ± 4

*Indicate significant (*p* < 0.05) differences between [Na]_pip_ groups within the same configuration.

### Facilitation of reverse mode further increases pacemaking

In the second set of AP measurements, we aimed to facilitate reverse NCX function via Na/K pump inhibition mediated Na^+^_i_ increase by using the perforated patch configuration. To inhibit Na/K pump, 1 µM strophantin was employed.

**Figure 7:**
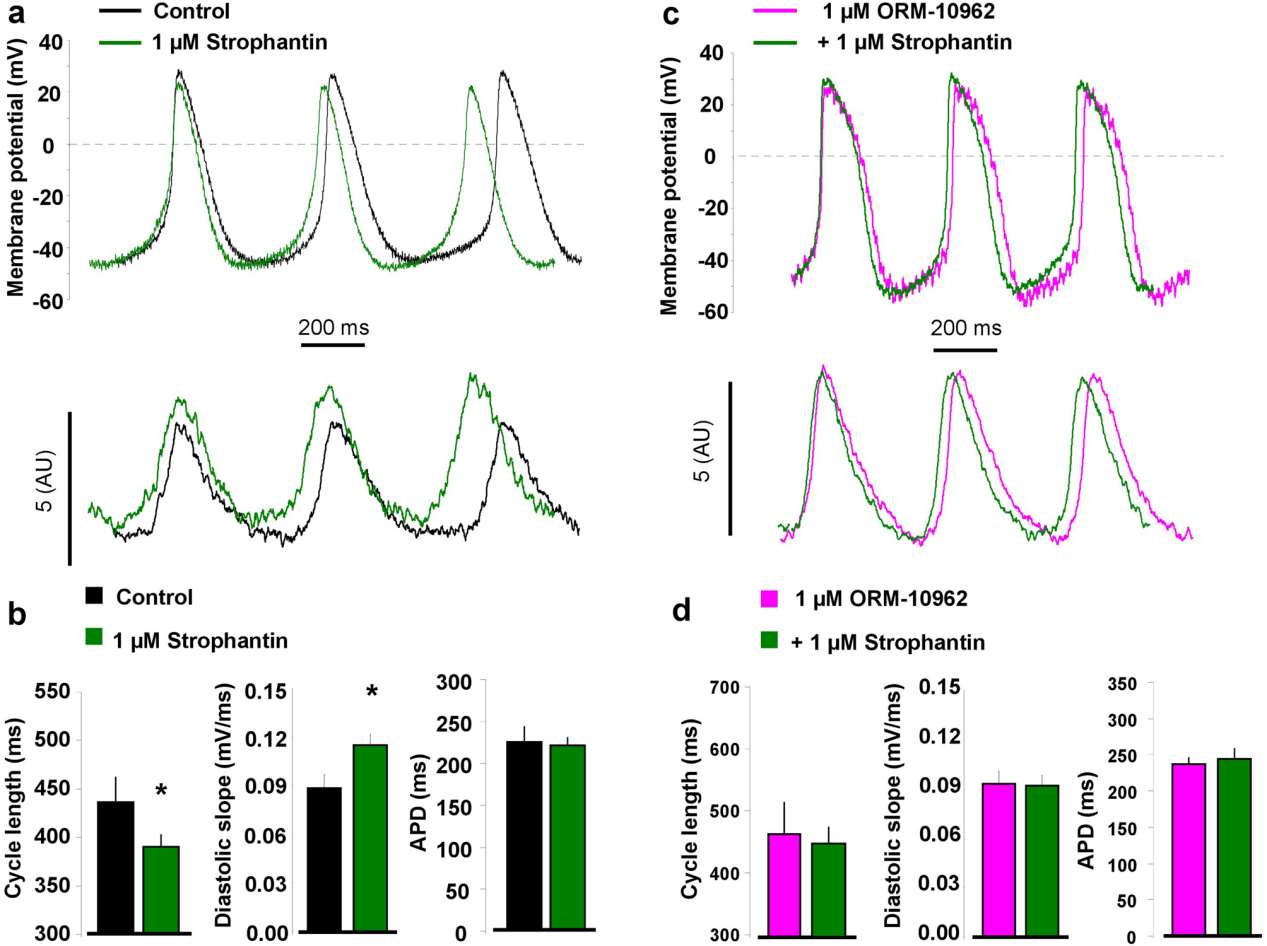
Evaluation of spontaneous AP and CaT characteristic measured by patch clamp technique in perforated cells. Panel **a** indicates that in the presence of 1 µM strophantin (green curve) both the AP cycle length (upper part) and the Ca^2+^-transient amplitude (lower part) was increased compared to control (black curve). Bar graphs illustrate statistically significant change of the cycle length and diastolic slope in the presence of strophantin (panel **b**). In contrast, when the cells were pre-treated with 1 µM ORM-10962 (panel **c**, pink curves) both the action potential cycle length (upper part) and the Ca^2+^ transient amplitude (lower part) remained unchanged. As bar graphs show none of the investigated parameters changed when the NCX was inhibited prior to strophantin employment (panel **d**). Data shown as mean ± SEM, n = 6, paired t-test, *means *p* < 0.05.

1 µM strophantin increased Ca^2+^_i_ by 18.9 ± 6% (n = 6). As a consequence, it shortened the cycle length (433 ± 25 ms vs 389 ± 11 ms, *p* < 0.05, n = 6, Fig. 7a–b) coupled with increased diastolic slope (0.09 ± 0.008 mV/ms vs 0.11 ± 0.006 mV/ms, *p* < 0.05, n = 6, Fig. 7a–b). The MDP (− 49 ± 2 mV vs − 54 ± 6 mV, n = 6) and APD (222 ± 18 ms vs 219 ± 10 ms, n = 6, Fig. 7a–b) remained unchanged (paired t-test).

In order to further challenge the role of reverse NCX in the strophantin effect, the exchanger was blocked by 1 µM ORM-10962 prior to strophantin administration. In this case, no change in the Ca^2+^_i_ level was observed. The AP parameters also exerted unaltered values (CL: 466 ± 50 ms vs 450 ± 26 ms; slope: 0.09 ± 0.01 mV/ms vs 0.08 ± 0.01 mV/ms; MDP: − 52 ± 3 mV vs − 46 ± 7 mV; APD: 236 ± 10 ms vs 244 ± 17 ms; n = 6, paired t-test, Fig. 7c-d).

### The Maltsev–Lakatta “minimal model” confirms an important role for Ca^2+^ influx through reverse NCX

Maltsev and Lakatta established a “minimal set” of sarcolemmal ion currents and intracellular Ca^2+^ clock components required for sinus node pacemaking in 2013 ^14^. The model includes only the I_CaL_ + I_Kr_ + I_NCX_ + Ca^2+^ clock components and omits I_CaT_, I_Ks_, I_to_, I_sus_, I_f_, I_st_, I_b(Na)_, I_Na/K_, I_b(Ca)_. A specific feature of this model is the prominent reverse NCX activity during the initial phase of the action potential.

**Figure 8:**
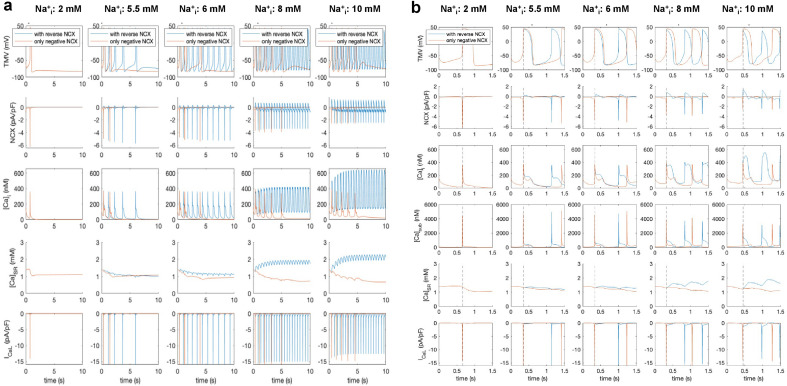
Computational simulation of Na-dependence of SN pacemaking by using the Maltsev–Lakatta minimal model^14^ in the presence (blue traces) and in the absence (brown traces) of reverse NCX. Brown traces represent NCX function when only the forward (negative current) mode was active. Intracellular Na^+^-concentration was changed from 2 to 10 mM (vertical columns) and the behaviour of the action potential (panel** a** first row), NCX-current (panel **a** second row), global intracellular Ca^2+^ (panel **a** third row), SR Ca^2+^ content (panel **a** fourth row) and I_CaL_ (panel **a** last row) were investigated. In the presence of reverse NCX (blue curves), increase of Na^+^_i_ enhances SN pacemaking with parallel increase of outward NCX and network SR Ca^2+^ content. In the absence of reverse mode, the SN pacemaking failed at all Na^+^_i_ levels (panel **a**) and a considerable loss in the SR Ca^2+^ content was observed.

This minimal model was used to assess the effects of Na_i_ changes on AP cycle length, intracellular Ca^2+^ levels, as well as NCX and I_CaL_ (Fig. 8). We found that in this minimal model with a reduced set of ionic currents and fixed Na_i_ and K_i_, at least 6 mM Na^+^_i_ is required for sustained pacemaking and that an increase of Na^+^_i_ from 6 to 10 mM decreases the AP cycle length (1011 ms → 450 ms, Fig. 8a first row) after transient changes had equilibrated. In line with this, a gradual increase of reverse NCX occurred (Fig. 8a second row, positive current), together with an increase of the Ca^2+^ transient amplitude and diastolic Ca^2+^ levels (Fig. 8a third row) as well as network SR Ca^2+^ levels (Fig. 8a fourth row) that are in agreement with our experimental observations.

It is important to note that the peak I_CaL_ magnitude gradually decreased as Na^+^_i_ increased (6 mM Na^+^_i_: − 16.5 pA/pF; 8 mM Na^+^_i_: − 15.1 pA/pF; 10 mM Na^+^_i_: − 12.6 pA/pF), however the carried charge, in contrast, increased (6 mM Na^+^_i_: − 4.8 pC; 8 mM Na^+^_i_: − 5.7 pC; 10 mM Na^+^_i_: − 6.9 pC; Fig. 8a last row). Table 2 summarizes the contributions of forward and reverse NCX as well as I_CaL_ to intracellular calcium cycling. The time spent in forward mode is 2.3–5.1 × longer than that spent in reverse mode and the charge carried is 2.5–3.1 × bigger. I_CaL_ carries 2.9–4.2 × more charge than reverse NCX but only 1.5–2.1 × more ions. The relative contribution of reverse NCX is bigger under high Na^+^_i_ conditions.

**Table 2:** Model results for varying levels of intracellular sodium.

[Na^+^]_i_ (mM)	6	8	10
cycle length (ms)	1011.9	455.9	451.4
tForward (ms)	846.9	319.5	335.4
tReverse (ms)	165.0	136.4	116.0
tForward/tReverse	5.1	2.3	2.9
QForward (fC)	3588.2	4623.7	5874.0
QReverse (fC)	1164.5	1752.4	2384.7
QForward/QReverse	3.1	2.6	2.5
QICaL (fC)	4845.4	5711.7	6944.0
QICaL/QReverse	4.2	3.3	2.9
nICaL/nReverse	2.1	1.6	1.5

Cycle length of spontaneous action potential (AP) initiation, time NCX spends in forward (tForward) and reverse (tReverse) mode during each AP, charge carried by NCX in forward (QForward) and reverse (QReverse) mode, carried by I_CaL_ (QICaL) and ratios between them including the ratio of ions carried by I_CaL_ (nICaL) and NCX in reverse mode (nReverse).

The role of reverse NCX in SN pacemaking was further elucidated by disabling reverse NCX in the model (setting NCX to zero whenever it was positive, brown traces in Fig. 8) and thus consider only forward NCX. A marked loss of SR Ca^2+^ content was observed (Fig. 8). Pacemaking could not be sustained without reverse NCX in the model. While pacemaking was observed for some beats for higher Na^+^_i_ levels, it ceased eventually (after < 10 s for all investigated Na^+^_i_ levels), suggesting that Ca^2+^ influx through reverse mode of the exchanger is essential for maintaining a stable SR Ca^2+^ level, or in other words, the Ca^2+^ influx provided by the I_CaL_ per se is insufficient to maintain pacemaking in this model.

## Discussion

In this study we characterized the reverse NCX and its functional role during the SN action potential. In accordance with previous numerical simulations^14^ (1) a voltage-, and Na^+^_i_ dependent outward current was found during the initial part of the SN action potential which was sensitive to ORM-10962 and NiCl_2_. (2) Higher SR Ca^2+^ content and (3) faster heart rate was observed in the presence of active reverse NCX.

### Does the reverse mode of NCX exist in SN cells?

The Na^+^/Ca^2+^ exchanger of the cardiac sarcolemma transporting 3 Na^+^ for 1 Ca^2+^ represents the main Ca^2+^ extrusion mechanism of the cell. The NCX exerts a thermodynamically defined reversal potential (E_NCX_) based on the transport stoichiometry: E_NCX_ = 3E_Na_ − 2E_Ca_. This means when the actual membrane potential (i.e.: action potential) is more positive than E_NCX_, Ca^2+^ entry and consequential outward current happens via the reverse mode of NCX. When membrane potential falls below E_NCX_, the direction of the transport changes to Ca^2+^ efflux and inward current is carried by forward mode.

Therefore, the presence or absence of reverse Na^+^/Ca^2+^ exchange in a given cell type is defined by the actual driving force of the NCX. Based on previous model simulations^14^, reverse operation of the NCX is expected when the membrane potential is more positive than 0 mV. When our experimental conditions were used for calculations to approximate the NCX equilibrium, the reverse mode was favoured in the first 55–65 ms from the AP upstroke (Supplementary Fig. 1a). In line with this calculation, application of NiCl_2_ dissected an outward current in this range when the reverse mode was active (i.e.: Na^+^_i_ was set to 8 mM in the pipette) but in the presence of 2 mM Na^+^_i_ the outward current was suppressed (Fig. 2). This result indicates that the reverse mode of the NCX could be able to develop under a SN AP, therefore the SN membrane potential theoretically enables operation of the reverse NCX.

In order to examine the development of reverse mode during working Ca^2+^-handling we also determined the NCX current as an ORM-sensitive current under canonical AP waveform as command potential (Supplementary Fig. 1b–c). Since the ORM-sensitive current is very susceptible to any spontaneous current change, we measured ORM-effects in separated groups to avoid any shortcomings from time dependent spontaneous decline of I_Ca_. The ORM sensitive current also revealed an outward current component at positive membrane potentials, when Na^+^_i_ was 8 mM.

Taking together, these results support previous modelling simulations^14^ suggesting an existing reverse NCX in SN cells by the following experimental reasons: (1) the Ni^2+^- and ORM-sensitive outward current component was only detectable in the presence of 8 mM pipette Na^+^ and disappeared when low Na^+^ was applied. This indicates that the outward component of the Ni^2+^- or ORM-sensitive current is Na^+^_i_ dependent. (2) As it was thermodynamically predicted, this outward component of the Ni^2+^- or ORM-sensitive current appeared only at positive membrane potentials, during the very first section of the AP.

How these findings in rabbit SN cells and models relate to human SN cells^21^ and models^22^^,^^23^ remains to be studied.

### Does reverse NCX activity modulate SN pacemaking?

Considering that SN pacemaking is critically based on the actual SR Ca^2+^ content, it may imply an important functional role of reverse NCX in cardiac SN pacemaking mechanism.

In line with these numerical calculations, in our experiments a larger CaT amplitude was found (seen in Fig. 3b) as a consequence of larger SR Ca^2+^ content in the presence of active reverse NCX (Fig. 4). Since the larger SR Ca^2+^ content generates larger Ca^2+^ release, NCX takes more time to extrude the Ca^2+^ providing longer decay in the case of 8 mM Na^+^_i_ group. However, this value did not reach statistical significance possibly due to the larger variance of the data. These changes could be attributable to the “extra” Ca^2+^ influx from the reverse exchange activity. The functional importance of reverse NCX was further explored during “recovery” of Ca^2+^ homeostasis after caffeine application (Fig. 5). The caffeine flush empties the SR, therefore reapplication of the stimuli, the Ca^2+^_i_ will be lower during the first steps (i.e.: “Ca^2+^ load”). Based on thermodynamical considerations, the lower Ca^2+^_i_ levels during the couple of first stimuli shifts the NCX equilibrium toward more negative values favouring an initially large, than gradually decreasing outward NCX parallel with the increase of Ca^2+^_i_. It is supposed that this initially large reverse component may boost up the recovery of CaT after caffeine application. As Fig. 5 illustrates, a step-by-step increasing diastolic Ca^2+^ level and CaT amplitude was found. Taken together these results, an important functional role of reverse NCX could be considered since: (1) the Ca^2+^ transient amplitude was higher in the presence of active reverse mode and (2) in line with this, the SR Ca^2+^ content increased. (3) The cells with active reverse exchange exerted faster refilling of Ca^2+^ in the initial steps after caffeine application, indicating synergistic effect between reverse NCX and I_Ca_ during SR Ca^2+^ loading.

The results of the experiments shown in Figs. 1, 2, 3, 4 and 5 indicated an existing reverse NCX that could provide additional Ca^2+^ influx to the cell in each cycle. Since SN pacemaking is largely based on Ca^2+^_i_, a potential role of reverse NCX in setting the actual SN AP cycle length was expected. When spontaneous APs were measured in the presence of 2 and 8 mM Na_pip_ we found shorter cycle length and steeper slope when reverse NCX was active. The parallel measured higher Ca^2+^ transient amplitude in this group indicates again that additional Ca^2+^ influx through active reverse mode facilitates pacemaking rate via shifting the Ca^2+^_i_ level (Fig. 6). Interestingly, the APD was also shortened in the presence of 8 mM Na_pip._ This could be a simple consequence of the higher Ca^2+^_i_-induced faster I_CaL_ inactivation, I_NaK_, and/or due to an additional repolarizing current via possible activation of the small-conductance Ca^2+^-activated K^+^-channel, as was demonstrated elegantly in a previous study^24^.

Computer simulations using the Maltsev–Lakatta minimal model suggest that spontaneous pacemaking fails at low Na_i_ levels (2 mM) and in the absence of reverse NCX (Fig. 8a,b panels). In contrast, spontaneous pacemaking was observed even in the 2 mM Na_pip_ group, i.e. with the inactive reverse NCX in our in vitro experiments. This discrepancy could be due to the fact that in the model the I_CaL_ current density gradually decreases as Na_i_ increases. Similarly, I_CaL_ integral is smaller when reverse NCX is absent in the model. Since in our experiments no Na^+^_i_ dependent I_CaL_ behaviour was observed (Supplementary Fig. 3), we suggest that Ca^2+^ influx through I_CaL_ did not change significantly and still provided sufficient loading for the SR in the experiments when reverse mode was suppressed by 2 mM Na_pip_.

Previous experiments with digoxigenin indicated that moderate increase in Na^+^_i_ shortens the cycle length parallel with increase of Ca^2+^ and arrhythmic behavior^25,26^. These results and our observations suggest that amplification of reverse NCX could further increase the spontaneous pacemaking. In our experiments, 1 µM strophantin was employed to increase Na^+^_i_ via Na/K-ATPase block. The increasing Na^+^_i_ is expected to shift E_NCX_ towards more negative values such that the reverse component of the NCX increases. In line with this, a higher pacemaking rate was found in response to strophantin administration (Fig. 7a). In contrast, the APD remained unchanged after strophantin. However, the Na/K pump generates net outward current, it is feasible that the increased Ca^2+^_i_ shortens the APD and these opposite effects lead to negligible change in the APD.

To further confirm the role of reverse NCX in this process, we inhibited the exchanger prior to strophantin administration. In the presence of NCX inhibition strophantin failed to alter the spontaneous automaticity, which could be the consequence of the unchanged intracellular Ca^2+^_i_ level after strophantin application in the presence of reverse NCX block (Fig. 7b). This result is in agreement with previous study on canine ventricular myocytes where strophantin-mediated spontaneous diastolic Ca^2+^ releases could be suppressed when the cells were previously treated with 1 µM ORM-10103^27^.

One can speculate that the observed results could be attributable to the NCX forward mode activity. Theoretically, the reduced Na_i_ (i.e. 2 mM Na_pip_) facilitates forward NCX leading to net Ca^2+^ loss while higher Na^+^-level inhibits the forward mode causing net Ca^2+^ gain. Therefore, in the case of 8 mM Na_pip_ both the Na^+^-mediated reverse mode activation and forward mode suppression could be able to increase the Ca^2+^_i_. However, in SN the forward mode activity has considerable contribution to the diastolic depolarization. Therefore, if Ca^2+^ increase was caused by forward mode inhibition the diastolic slope would decrease as an indicator of inward current reduction.

Numerical simulations indicated progressively increasing heart rate as Na^+^_i_ increases. As Fig. 8 illustrates, both reverse NCX and the charge carried by I_CaL_ are increased and could account for the accelerated pacemaking. However, as Fig. 8 demonstrates, the suppression of the reverse component causes a great loss in the SR Ca^2+^ content and abrupt termination of spontaneous pacemaking. Obviously, the charge carried by I_CaL_ also reduced in this case, but it may imply that Ca^2+^ influx by the reverse activity contributes in setting the SR Ca^2+^ content, suggesting important indirect role in setting the actual heart rate. This is underpinned by the fact that SR Ca^2+^ loss and failure of automaticity could be rescued by improved SERCA activity (Supplementary Fig. 4). This result may further support that Ca^2+^ influx through reverse exchange provides a functionally important fraction of the total SR Ca^2+^ content, i.e. the reverse NCX may indirectly contribute to fine tuning of the heart rate.

### Is the reverse NCX more important in SN than in ventricular cells?

The exact role of the reverse NCX in *ventricular* myocytes is not fully clarified under normal circumstances. Initial studies suggested that reverse mode could trigger Ca^2+^ release^28,29^ however this was later questioned^30^. Further studies claimed that reverse Ca^2+^ influx is able to augment the Ca^2+^ transient via a synergistic interaction with the I_CaL_ that may depend on the actual Na^+^_i_ level^31–35^. Based on these experimental results, the reverse mode in ventricular myocytes is expected to facilitate the Ca^2+-^induced Ca^2+^ release by improving its efficacy however it seems no essential for the normal Ca^2+^ handling.

The Ca^2+^ influx of the reverse NCX and I_CaL_ can be calculated and compared between ventricular and SN myocytes using respective computational models. For the Mahajan rabbit ventricular myocyte model^36^ (dynamic Na^+^_i_ ranging between 11.4 and 11.5 mM in silico; in vitro range: 9–11.5 mM^37^), the I_CaL_/reverse NCX Ca^2+^ influx ratio is 17.2 compared to 3.3 found in this study for SN cells (at Na^+^_i_ of 8 mM) under in silico condition, and 2.81 under in vitro condition.

This large discrepancy between ventricular myocytes and SN cells could be the consequence of the long plateau phase in the ventricular cells providing long-lasting opening of the I_CaL_ and a maintained Ca^2+^ influx. In SN cells, the absence of the plateau phase considerably restricts the I_CaL_ opening time limiting I_CaL_ Ca^2+^ influx. This may explain that under normal condition, in the ventricular myocytes, the I_CaL_ is markedly dominating over the reverse NCX but it may represent an important source of Ca^2+^ influx in SN cells since the Ca^2+^ entry via I_CaL_ is restricted due to the characteristic SN AP waveform.

### Electrical heterogeneity within the SN

There is a large body of evidence that the SN exerts considerable electrical heterogeneity (reviewed by:^38^). The action potentials recorded from the central region markedly differ from the action potentials obtained from the transitional or peripheral zone^39^. In context of the reverse mode, two important differences should be mentioned: from the centre region to the distal areas the (1) action potential upstroke becomes significantly larger and (2) the cycle length shortens. These differences could be explained by the influence of the atrial muscle^40^, the higher role of I_f_ in the periphery^41,42^, and the different role of the I_CaL_ in pacemaking from the centre to the periphery^39,43^.

Considering the results of this study, it is feasible that the small upstroke in the centre region considerably restricts the reverse function, while the distal areas may enable increasing amount of reverse mode. This increasing reverse NCX gradient from the centre to the periphery may contribute in the observed AP cycle length shortening in the transitional zone and the periphery, therefore the reverse NCX could contribute to the electrophysiological heterogeneity of the SN.

## Conclusion

In this study, we identified a Na^+^- and voltage-dependent, ORM-10962 and Ni^2+^-sensitive outward current component appearing during the early SN action potential that could be considered as reverse NCX.

Our results suggest that Ca^2+^ influx through the reverse NCX may contribute to the setting of the SR Ca^2+^ content and might therefore represent an additional mechanism of the SN coupled-clock pacemaking system that contributes to the control of the heart rate in SN cells.

## Study limitations

1. It is important to note that ORM-sensitive currents (in Supplementary Fig. 1) do not allow to calculate current parameters (amplitude, time, carried charges) due to 2 reasons: (a) 1 µM ORM-10962 only partially inhibits the NCX. (b) ORM-sensitive current is very susceptible to any spontaneous current change, thus we measured ORM-effects in separated groups to avoid any shortcomings from time dependent spontaneous decline of I_Ca_. Considering these limitations, the aim of the ORM-sensitive current was to demonstrate the existence of the reverse NCX current during the initial phase of the AP.
2. In the experiments, we used only one canonical action potential waveform. Since the SN is a heterogeneous system, our results can be interpreted as representative for SN cells that have a relatively large action potential overshoot and should not be generalized to the entire SN.
3. A possible contribution of the I_Na_ may change the intracellular Na^+^-level of the cells that could influence the function of the NCX.
4. In the experiments, we used 2 and 8 mM NaCl in the patch pipette. This change in the intracellular Na^+^ level could also influence the I_Na_, Na/K pump, Na/H pump and I_f_.

## Supplementary Information

Supplementary Information 1.

Supplementary Information 2.

Supplementary Information 3.

Supplementary Information 4.

Supplementary Information 5.

## Data Availability

All data generated or analysed during this study are included in this published article [and its supplementary information files].
