# Supplementary figures and images for: The reverse mode of the Na+/Ca2+ exchanger contributes to the pacemaker mechanism in rabbit sinus node cells
Source: Sci Rep. 2022 Dec 17;12:21830. doi: 10.1038/s41598-022-25574-8 (PMC9759562; doi:10.1038/s41598-022-25574-8)

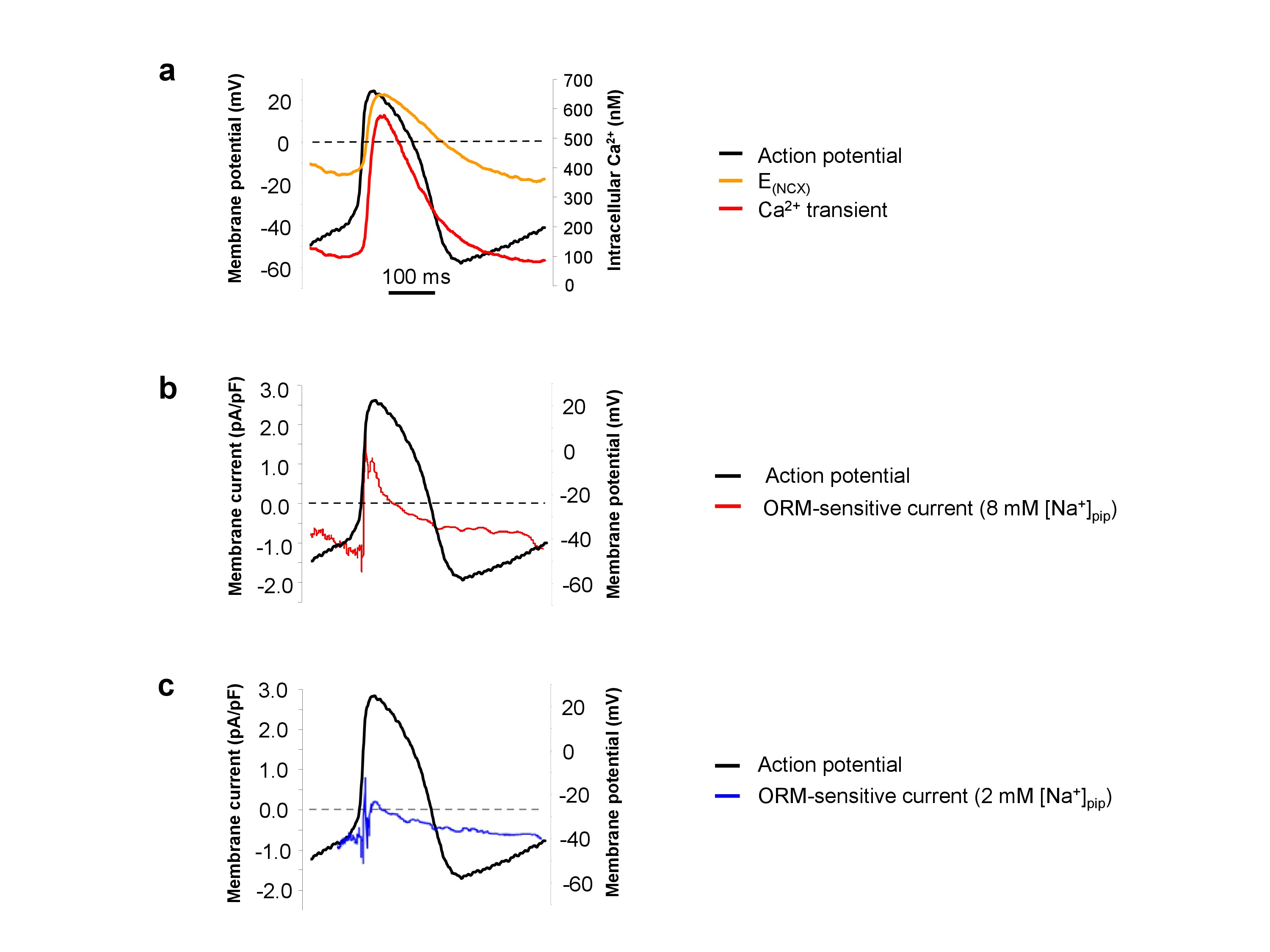

Supplement: Supplementary file 1 — Supplementary Information 1. [file 41598_2022_25574_MOESM1_ESM.jpg]

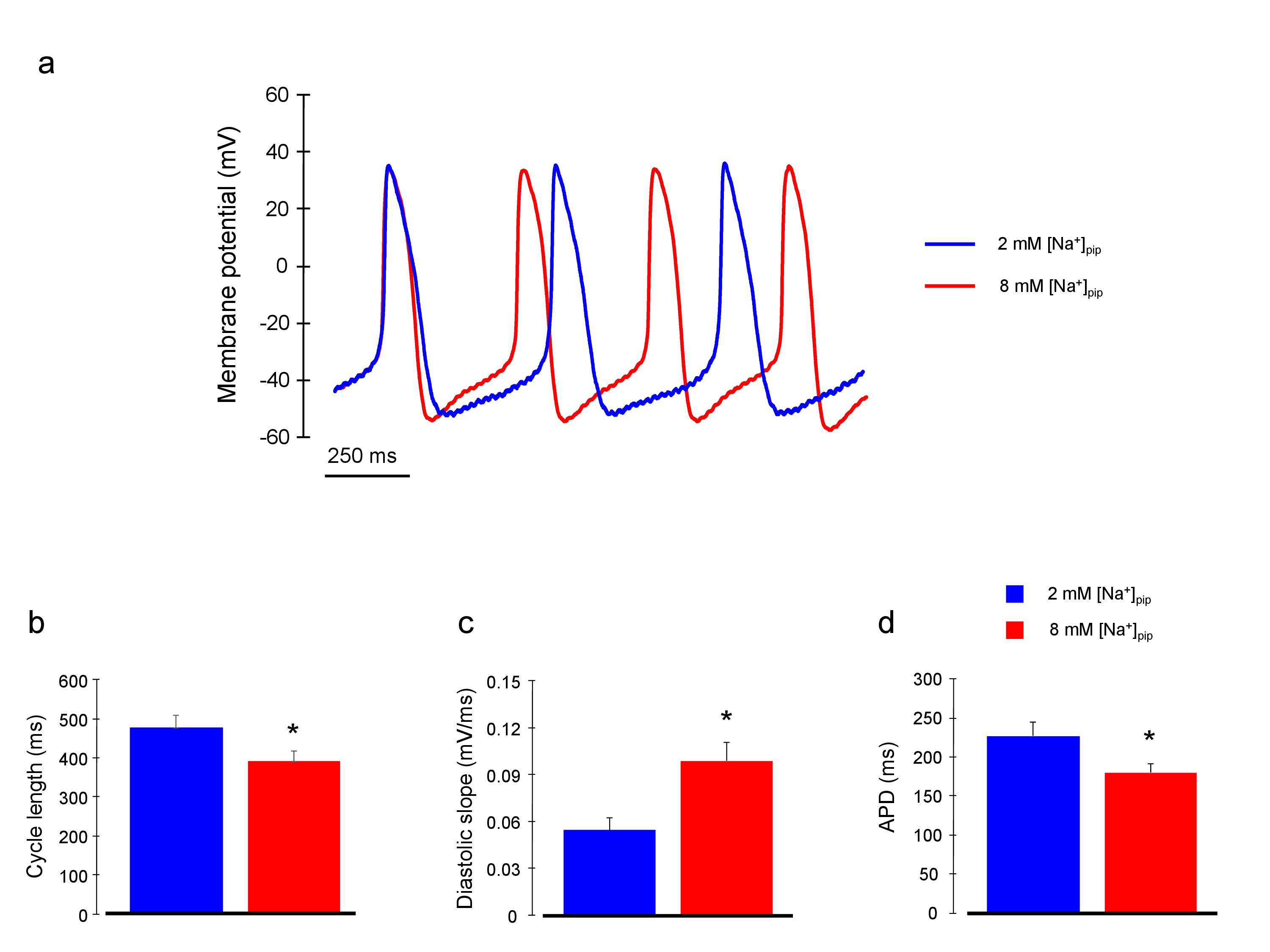

Supplement: Supplementary file 2 — Supplementary Information 2. [file 41598_2022_25574_MOESM2_ESM.jpg]

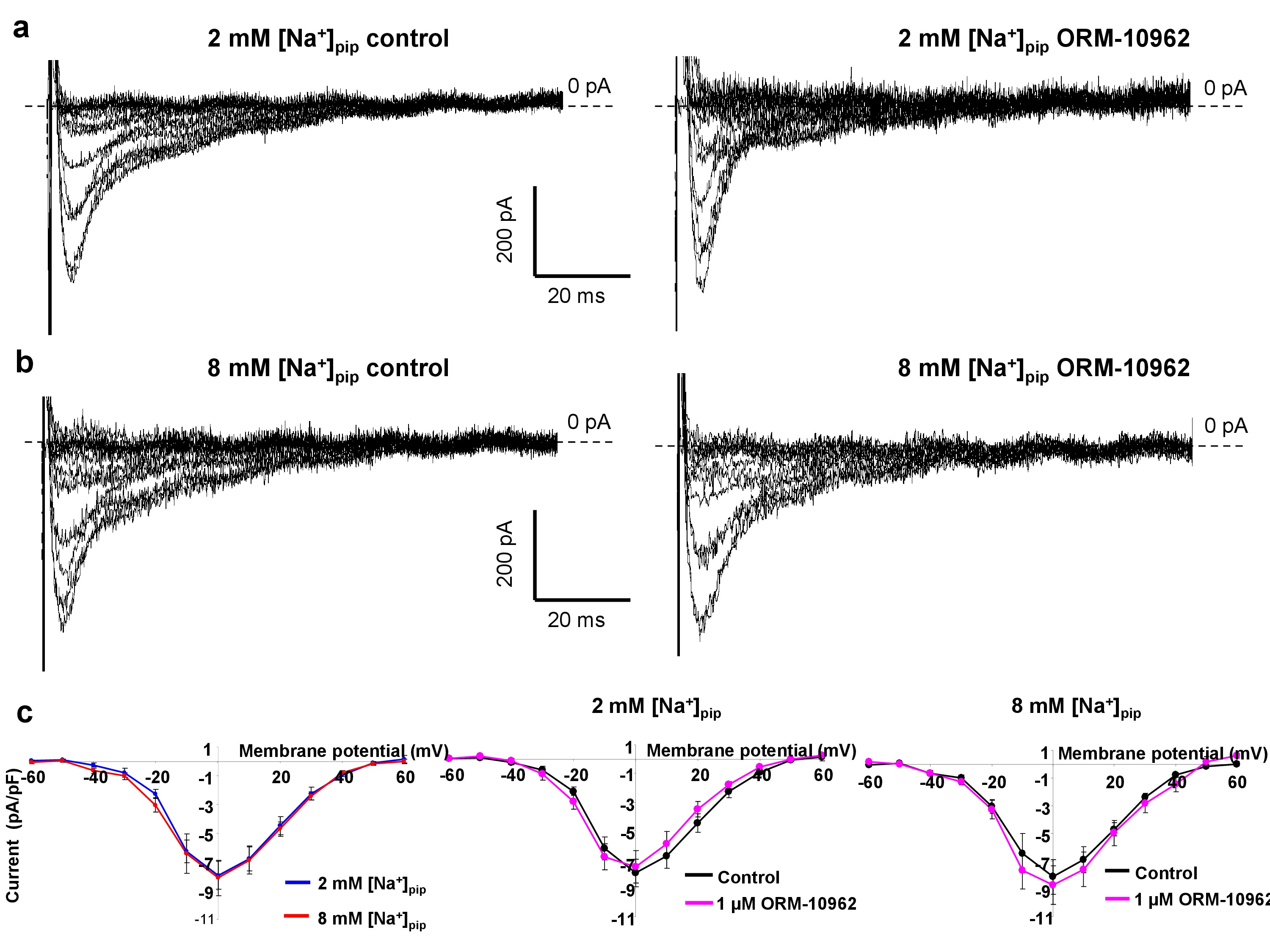

Supplement: Supplementary file 3 — Supplementary Information 3. [file 41598_2022_25574_MOESM3_ESM.jpg]

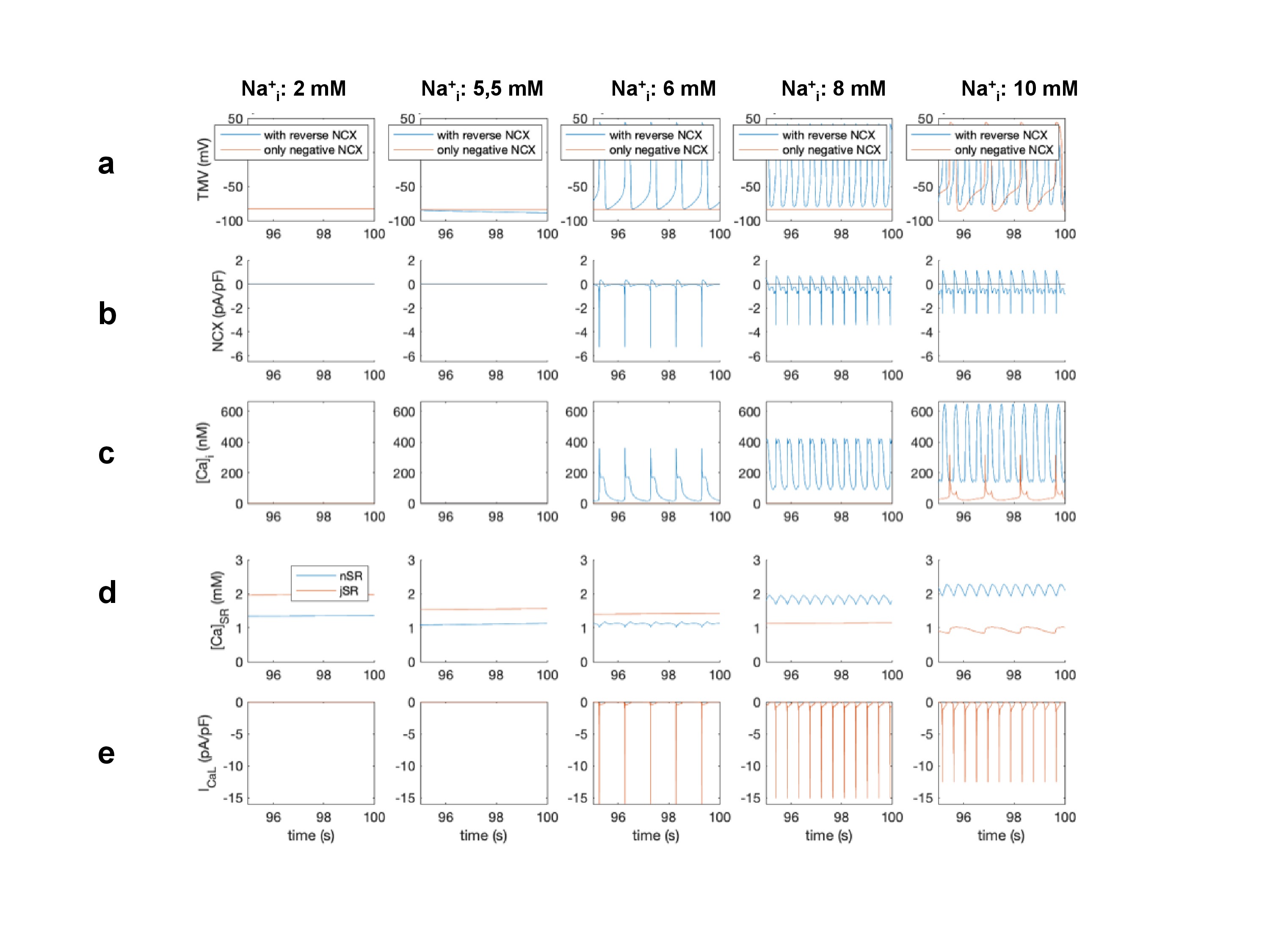

Supplement: Supplementary file 4 — Supplementary Information 4. [file 41598_2022_25574_MOESM4_ESM.jpg]
