# Supplementary material for: The reverse mode of the Na+/Ca2+ exchanger contributes to the pacemaker mechanism in rabbit sinus node cells
Source: Sci Rep. 2022 Dec 17;12:21830. doi: 10.1038/s41598-022-25574-8 (PMC9759562; doi:10.1038/s41598-022-25574-8)
Supplement: Supplementary file 5 — Supplementary Information 5. [file 41598_2022_25574_MOESM5_ESM.docx]

- Supplementary material -

**The reverse mode of the Na^+^/Ca^2+^ exchanger contributes to the pacemaker mechanism in rabbit sinus node cells**

*Reverse NCX in spontaneous automaticity*

Noémi Tóth^a^, Axel Loewe^b^, Jozefina Szlovák^a^, Zsófia Kohajda^c^, Gergő Bitay^a^, Jouko Levijoki^d^, Julius Gy. Papp^a,c^, András Varró^a,c^, Norbert Nagy^a,c^

^a^Department of Pharmacology and Pharmacotherapy, Albert Szent-Györgyi Medical School, University of Szeged, Hungary

^b^Institute of Biomedical Engineering, Karlsruhe Institute of Technology (KIT), Karlsruhe, Germany

^c^ELKH-SZTE Research Group of Cardiovascular Pharmacology, Szeged, Hungary

^d^Orion Pharma, Espoo, Finland

**Supplementary results**

***Calculations of NCX equilibrium potential***

We calculated the NCX equilibrium potential (E_NCX_, **Supplementary Figure 1a**, orange trace) related to the corresponding canonical action potential voltage command (**Supplementary Figure 1a**, black trace) to estimate the thermodynamically favourable time frame for reverse mode development. A typical Ca^2+^ transient waveform from the 8 mM [Na]_pip_ group was used for calculation (**Supplementary Figure 1a**, red trace). As superimposed traces indicate, during the first section of the action potential the E_NCX_ is more negative than the action potential enabling the development of reverse exchange function.

**Supplementary Figure 1b** illustrates the ORM-sensitive current defined by application of 1 μM ORM-10962 in the presence of 8 mM [Na]_pip_. The control and ORM-treated curves were recorded in different cells to avoid I_CaL_ run-down mediated distortion of the current. The reversal of the ORM-sensitive current occurred at a similar phase of the action potential as the E_NCX_ predicts. In contrast, when [Na]_pip_ was set to 2 mM, the outward component of the ORM-sensitive current was suppressed (**Supplementary Figure 1c**). These results suggest the presence of a voltage- and Na^+^-dependent ORM-sensitive current during the SN AP.

***Measurements of action potentials by perforated patch clamp technique***

The patch pipette solution contained (in mM): 120 K-gluconate, 2.5 MgATP, 2.5 K_2_ATP, 5 HEPES, 20 KCl supplemented with 2 mM NaCl or 8 mM NaCl accordingly to 2 mM [Na]_pip_ and 8 mM [Na]_pip_ groups, titrated to pH 7.2 with KOH. 35 µM β-escin was added to the pipette solution to achieve the membrane patch perforation. Since the pipette solution was identical, perforated and whole cell recordings were comparable.

In line with whole cell experiments, the 8 mM [Na]_pip_ group exerted shorter cycle length (392±25 ms vs 478±31, p<0.05, n=9 and 8 respectively, **Supplementary Figure 2** **panel a and b**), steeper diastolic slope (0.098±0.01 mV/ms vs 0.05±0.01 mV/ms, p<0.05, n=9 and 8 respectively, **Supplementary Figure 2 panel c**) and longer APD (179±11 ms vs 226±17 ms, p<0.05, n=9 and 8 respectively, **Supplementary Figure 2 panel d**). No change was detected in the MDP between 8 and 2 mM [Na]_pip_ groups (-53±4 mV vs -52±3 mV, p=0.9, n=9 and 8 respectively).

***Comparison of Ca^2+^-currents***

A composite Ca^2+^-current was measured from a holding potential of -60 mV to 60 mV with increments of 10 mV. The intracellular milieu of the cell was buffered with 10 mM EGTA. The external solution contained in mM: 135 NaCl, 10 CsCl, 0.33 NaH_2_PO_4_, 10 TEACl, 1 MgCl_2_, 10 glucose, 10 HEPES, 1 CaCl_2_, 0.2 BaCl_2_, 20 µM ouabain, 50 µM lidocain and titrated to pH 7.4. The internal solution contained in mM: 125 CsCl, 20 TEACl, 5 MgATP, 10 HEPES, 10 EGTA, titrated to pH 7.2 with CsOH. As original traces (**Supplementary Figure 3a** and **b)** and current-voltage diagrams (**Supplementary Figure 3c)** demonstrate no alterations occurred between control currents (**Supplementary Figure 3c, left panel**), and the ORM effects in both [Na]_pip_ groups (**Supplementary Figure 3c, middle and right panels).**

The L-type Ca^2+^ current was also measured under canonical AP waveform as command potential in order to assess the carried charges within one AP cycle. The external solution contained (in mM): 135 NaCl, 10 CsCl, 0.33 NaH_2_PO_4_, 10 TEACl, 1 MgCl_2_, 10 glucose, 10 HEPES, 1.8 CaCl_2_, 0.2 BaCl_2_, 20 µM ouabain, 50 µM lidocain and titrated to pH 7.4. The internal solution contained (in mM): 125 CsCl, 20 TEACl, 5 MgATP, 10 HEPES. Note that the T-type Ca^2+^ channels were suppressed during these experiments. We also aimed to reduce the NCX as possible by using 10 mM EGTA (in the pipette), 0 mM intracellular Na^+^, and by 1 µM ORM-10962. The data was collected from 6 hearts, the carried charges were found: 5.9±2 pC.

***Improved SERCA function restored pacemaking in the absence of reverse NCX***

The failure of pacemaking without reverse NCX (**Figure 8, brown traces**) could be reverted by increasing the SERCA activity (*P_up_*) 1.5-fold in the presence of 10 mM Na^+^_i_ (**Supplementary Figure 4**). Under these conditions, the model exerted slow but maintained pacemaking with stable SR Ca^2+^ content suggesting that improved SERCA function may partially compensate for the Ca^2+^ loss caused by non-functional reverse NCX. The Na-dependence of this effect could be attributed to the fact that the largest I_CaL_ integral was observed in the presence of 10 mM Na^+^_i_. These results further support that reverse NCX and I_CaL_ synergistically refuel the Ca^2+^ clock.

**Supplementary Figure legends**

**Supplementary Figure 1**: Comparison of NCX reversal potential (E_NCX_, **panel a**, orange trace) with ORM-sensitive currents (**panel b**, red trace and **panel c**, blue trace).

**Supplementary Figure 2:** Measurements of SN AP by using perforated patch configuration. The patch pipette contained 2 and 8 pipette NaCl. **Panel a** shows representative AP traces, while bar graphs indicate cycle length (**b**), diastolic slope (**c**) and APD (**d**). * means p<0.05, independent t-test.

**Supplementary Figure 3**: Investigation of Ca^2+^ currents in SN isolated cells. **Panel a** and **b** illustrate representative original current traces demonstrating Ca^2+^ currents in the absence (left) and in the presence (right) of 1 μM ORM-10962 in 2 mM (**panel a,** n=4-4) and 8 mM (**panel b,** n=5-5) pipette NaCl groups. Comparisons of current-voltage relationships of I_Ca_ between 2 and 8 mM NaCl_pip_ (left), control and ORM in 2 mM [Na]_pip_ (middle), and control and ORM in 8 mM [Na]_pip_ exert no change between groups. Independent t-test.

**Supplementary Figure 4**: Improved SERCA function rescues the SN from pacemaking failure in the absence of reverse NCX function. In this set of modelling, we increased the SERCA function with 50%. When Na^+^_i_ was set to 10 mM we found that SR Ca^2+^ level was able to reach the critical level required for pacemaking even if the reverse mode was blunted (blue trace, right column). This result may further indicate the important role of reverse Ca^2+^ influx via the exchanger to load the SR Ca^2+^ content.
